# Hyperlipidemia: A Review of the Novel Methods for the Management of Lipids

**DOI:** 10.7759/cureus.16412

**Published:** 2021-07-15

**Authors:** Kosisochukwu J Ezeh, Obiora Ezeudemba

**Affiliations:** 1 Internal Medicine, Jersey City Medical Center, Jersey City, USA; 2 Internal Medicine, St. Vincent's Medical Center, Bridgeport, USA

**Keywords:** pcsk-9 inhibitor, lipid metabolism, statin-induced myopathy, bempedoic acid, niacin, ezetimibe, ldl-c, low density lipoprotein-cholesterol

## Abstract

Hyperlipidemia is the most common modifiable cause of atherosclerotic cardiovascular disease. Our understanding of managing hyperlipidemia has led us to the concept of the inverse correlation of low-density lipoprotein cholesterol (LDL-C) and non-high-density lipoprotein (non-HDL) cholesterol with the advent of a major adverse cardiovascular event. This review will provide an overview of lipids and their metabolism. Additionally, it will focus on hyperlipidemia and approaches to its management.

## Introduction and background

Hypercholesterolemia and hypertriglyceridemia contribute to the development of atherosclerosis. Atherosclerosis is a health condition strongly associated with ischemic heart disease (IHD) [1]. Cardiovascular disease (CVD) is the number one cause of mortality worldwide. Therefore, hyperlipidemia treatment plays a vital role in managing coronary artery disease (CAD) patients or those at increased risk of CAD worldwide. Lipids have numerous imperative and diverse responsibilities as they are the essential building blocks of body cells [2]. Hyperlipidemia is a health condition that increases plasma lipids and lipoproteins. Some examples of plasma lipids are cholesterol, triglycerides (TGs), phospholipids, and cholesterol esters. On the other hand, plasma lipoproteins include very-low-density lipoprotein (VLDL), low-density lipoprotein (LDL), and reduced high-density lipoprotein (HDL) levels. Lipoproteins are macromolecules comprising lipids and proteins. Their structure enables the lipids to combine well with other aqueous body fluids. They are categorized into non-polar lipids, polar lipids, and specific proteins. Non-polar lipids include cholesteryl esters and TGs while polar lipids include unesterified cholesterol and phospholipids. The specific proteins are also known as apolipoproteins. Apolipoproteins are amphiphilic proteins that bind to both lipids and the plasma [3]. Lipoproteins are also classified according to their densities. There are HDL and non-HDL such as chylomicrons (CM), VLDL, LDL, and intermediate-density lipoproteins (IDL).

## Review

Lipid metabolism begins with the hydrolysis of a vast percentage (approximately 50-80%) of main TGs in the presence of lipoprotein lipase. Lipoprotein lipase is found at the endothelial site of several peripheral tissues. On the contrary, TGs are present in the residue particles like cholesteryl esters and most lipid-soluble vitamins. Insulin found in the adipose tissue activates the lipoprotein lipase. The enzymic insulin activity is hence amplified after a person consumes a meal rich in carbohydrates and fats. The alignment of these fragments is also changed since CM atoms obtain cholesteryl esters. The modification occurs due to the transfer of cholesteryl esters from cholesterol-rich lipoproteins, a procedure facilitated by the cholesteryl ester transfer protein (CETP). Formerly obtained apolipoprotein E (apo E) enables the liver and other tissues to uptake hepatocytes (the residues). After the liver's uptake, there is repackaging or metabolization of lipid components of the remnants into VLDLs. VLDLs experience an intravascular metabolism impartially analogous to that of CMs. CM intravascular metabolism pertains to acquiring transferrable apoproteins, CETP-facilitated acquisition of cholesteryl esters, hydrolysis of a considerable portion of TGs, and forming residue particles known as IDLs.

There is a removal of approximately half of the IDLs, which are later endocytosed (primarily in the liver). The remaining portion of IDLs undertakes further lipolysis of TGs and phospholipids and converts them into LDLs rich in cholesterol. LDL units mainly transfer cholesterol and phospholipids (the cell membranes' main fat components) that facilitate peripheral tissue cells' regeneration. Endocytosis facilitates the removal of LDL atoms from the flow after binding to a definite LDL receptor (LDL-R). The LDL-R distinguishes apoproteins B and E. Endocytosis mainly occurs in the liver under the control of regulatory mechanisms that prevent cellular buildup of cholesterol. Notably, LDLs can effortlessly cross the endothelium through a procedure called transcytosis. Besides, at least 25-30% of LDLs present themselves in blood vessel walls' sub-endothelial space. Nonetheless, LDL reverses intact into the circulation [3]. In the intima, free radicals formed by local macrophages attack LDL, forcing it to endure peroxidative damage. The altered atoms are then phagocytosed by macrophages via scavenger receptors facilitating cholesterol buildup and foam cell development. Moreover, oxidized LDL is cytotoxic and triggers soreness within the arterial wall. The inflammatory reaction is also known as an atherosclerotic lesion, and it relates to endothelial dysfunction. The endothelial dysfunction triggers nitric oxide (NO) release, production of adhesion molecules, and propagation of smooth muscle cells. The apo-A-1 produced in the intestines and the liver combines with phospholipids to form HDLs. The HDL loads extra saturated fat existing in extrahepatic cells and tissues [via the adenosine triphosphate (ATP)-binding cassette A1 system] and transfer it back to the liver in a process known as reverse cholesterol transport [3].

Classification of hyperlipidemia

Hyperlipidemia can be broadly classified as either primary or secondary. Primary hyperlipidemia could be divided into the isolated elevation of cholesterol, isolated elevated TG, and elevations of both. The leading cause of primary hyperlipidemia is the genetic formulation, environmental factors, or both. The table below shows a list of genetic-related hyperlipidemia [4] (Table 1). People with genetic syndrome show incredibly high cholesterol and TG levels over 300 mg/dL and 500 mg/dL. They also show xanthomas, robust genetic history of hyperlipidemia, or primary CVD. Lastly, they show the absence of a predictable response to maximum therapeutic doses of lipid-lowering agents. The following table (Table 1) shows some of the forms of primary hyperlipidemia and cholesterol and triglyceride composition that contribute to their development.

**Table 1:** Forms of primary hyperlipidemia and cholesterol and triglyceride ApoC-II: apolipoprotein C-II

Isolated cholesterol elevation	Cholesterol and triglyceride elevation	Isolated triglyceride elevation
Genetic familial hypercholesterolemia	Combined familial hyperlipidemia	Lipoprotein lipase deficiency
Familial defective apolipoprotein B100	Familial dysbetalipoproteinemia (type III)	ApoC-II deficiency (deficiency of lipoprotein lipase activation)
Elevated plasma lipoproteins	Hepatic lipase deficiency	Familial hypertriglyceridemia
Polygenic hypercholesterolemia	Coronary heart disease	Coronary heart disease
Cerebrotendinous xanthomatosis	Cerebral cholesterosis	Cerebral cholesterosis
Sitosterolemia	Beta-sitosterolemia plant sterol storage disease	Phytosterolemia

Secondary hyperlipidemia emanates from components of other factors such as drugs, diet, or existing illnesses. The table below (Table 2) shows specific causes and causative components of secondary hyperlipidemia.

**Table 2:** Causes of secondary hyperlipidemia HIV: human immunodeficiency virus; PCOS: polycystic ovary syndrome

Secondary cause	Causative agents/components
Drugs	Diuretics (thiazide), glucocorticoids, sex hormones, beta-blockers, antipsychotics, antiretrovirals, immunosuppressants, retinoic acid derivatives
Diet	Saturated and trans fat, high-sugar beverages and foods, whole milk, red meat, alcohol, excess calories
Diseases	Type 2 diabetes, metabolic syndrome, obesity, hypothyroidism, HIV, PCOS, renal disease (nephrotic syndrome)

Hyperlipidemia management 

The relationship between hypercholesterolemia and coronary heart disease (CHD) has been well established, and for decades, the practice has been to decrease LDL cholesterol (LDL-C) in the blood. The positive results of the Lipid Research Clinics Coronary Primary Prevention Trial (LRC-CPPT) led to the establishment of this practice. It has proven beneficial in minimizing LDL-C levels in a statistically substantial way in cardiovascular actions. However, its success depends on relying on a strictly low-fat diet and cholestyramine for men battling hypercholesterolemia. After the positive results of the LRC-CPPT, hydroxymethyl glutaryl coenzyme A reductase inhibitor medications (primarily statins) became available. The combination of these treatment options led to a significantly better LDL-C drop with scarce negative side effects. The outcomes of a sequence of statin-induced cholesterol-lowering trials have shown that the nationwide treatment for hyperlipidemia should adopt a new LDL-C objective of less than 70 mg/dL [5]. Most of the hyperlipidemia patients who have consumed cholesterol-lowering pills have successfully attained lower LDL-C values. Below are some of the approaches to managing hyperlipidemia.

Statins

Statins prevent the 3-hydroxy-3-methylglutaryl coenzyme A (HMG-CoA) reductase enzyme. HMG-CoA is an enzyme that controls the rate of cholesterol biosynthesis in the body. Statins act by lowering LDL-C and TGs and slightly elevating HDL levels, thereby providing intermediate care for dyslipidemia management. Unfortunately, statins have several side effects, such as liver damage, type 2 diabetes mellitus, and muscle pain. Fortunately, the benefit (dyslipidemia management) outweighs statin-related risks. Statins avert smooth muscle cell relocation and spread and obstruct the stimulation of tumor necrosis factor-alpha (TNF-alpha), interleukin 1 (IL-1) beta, and other interleukins, which contribute to inflammation, a common symptom of hyperlipidemia.

Bempedoic Acid

The United States Food and Drug Administration (FDA) approved Esperion's bempedoic acid (Nexletor) (Esperion Therapeutics, Ann Arbor, MI) tablet as a remedy for lowering LDL-C in February 2020 [6]. Nexletor is an LDL-C-lowering oral tablet that has to be consumed daily and has no statins.

It acts by activating the bempedoic acid in the liver through an extensive chain of acyl-CoA synthetase-1 (ACSVL1) to hinder ATP-citrate lyase (ACL) and acts as an upstream of HMG-CoA reductase. The upstream action helps to upregulate LDL-R; thus, it intensifies the clearance of LDL-C. Also, bempedoic acid is effective for people allergic to statin therapy because it prevents myalgias (one of the probable side effects of statin treatment). Notably, activated bempedoic acid is absent in the skeletal muscle though it functions similarly to statins in the cholesterol synthesis pathway.

Ezetimibe

Ezetimibe is also effective in the management of hyperlipidemia. It selectively constrains the small intestines from absorbing cholesterol, and thereby reduces the delivery of intestinal cholesterol to the liver by obstructing the Niemann-Pick C1-Like 1 (NPC1L1) protein (a sterol transference protein in the human body) [7]. It also averts the absorption of bile acid in the small intestine, depresses LDL, upsurges HDL slightly, and substantially lowers TGs. Nevertheless, ezetimibe leads to side effects such as abdominal pain and myalgia [8].

PCSK9 Inhibitors

**Figure 1:**
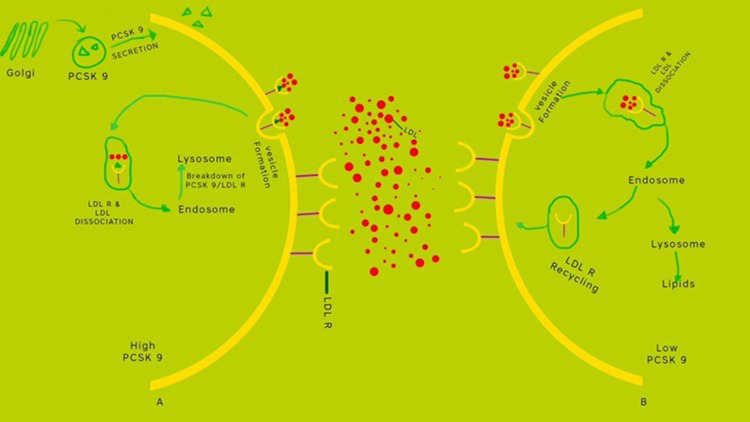
Mechanism of PCSK9 PCSK9: proprotein convertase subtilisin/Kexin type 9

Another approach to manage hyperlipidemia is through the release of proprotein convertase subtilisin/Kexin type 9 (PCSK9). PCSK9 is an enzyme programmed by the PCSK9 gene found on chromosome 1 of the human body. Hepatocytes produce PCSK9. The figure below (Figure 1) demonstrates how PCSK9 regulates LDL receptors. The higher the level of PCSK9, the lower the LDL-R's expression in the cell surface, and hence the higher LDL-C.

With the growing knowledge about PCSK9, it has been perceived that various methods can inhibit its action, such as monoclonal antibodies, small interfering RNA (siRNA), vaccines, antisense oligonucleotides, small molecules, mimetic peptides, and adiponectin. Many of these are currently in phase 1 trials, except monoclonal antibodies, small interfering RNA (inclisiran), which are in phase 3 (ORION-4), and antisense oligonucleotides, the trial of which was terminated following severe renal adverse effects [9].

The application of monoclonal antibodies has been the most effective approach to constrain PCSK9 and reduce LDL levels in the body. Notable drugs in this class include bococizumab, alirocumab (Praluent), and evolocumab (Repatha). Further, the ODYSSEY OUTCOMES trial has shown that alirocumab meets its primary endpoint, such as major adverse cardiac events (MACE). On the other hand, the Fourier trial showed evolocumab meeting its prime endpoint, including CV death, stroke, myocardial infarction (MI), and hospitalization for uneven angina.

Another approach to PSCK9 inhibition is the use of siRNAs. These constitute about 20-30 nucleotides found in the RNA molecules. Recently, they have been shown to have critical regulation and expression functions of eukaryotic genomes. They achieve it by interfering with the specific genes' appearances that are complemented by nucleotide series. They affect the deprivation of messenger RNA (mRNA) post-transcription, thereby averting transformation.

A good example is inclisiran, which is a terminal-acting, synthetic siRNA. It is directed against PCSK9 that targets the triantennary N-acetylgalactosamine carbohydrates. These carbohydrates bind to profuse liver-expressed asialoglycoprotein receptors, triggering an uptake of inclisiran precisely into the hepatocytes. Afterward, the siRNA molecules follow the ordinary RNA interference trail (RNAi) by binding themselves between cells up to the RNA-induced silencing complex (RISC). This action enables the siRNA to slice the mRNA molecules precisely, thus encoding PCSK9. Further, adnectins/monobodies and small peptides have indicated promising results in the inhibition of PCSK9, and thereby reducing LDL in phase I scientific experiments. A hyperlipidemic patient showed a 48% chance of reduced LDL-C in the body. The main benefit of adnectins is their small size compared to monoclonal antibodies; hence, they are easier and cheaper to produce. All the above PCSK9 mechanisms operate in the same way to regulate LDL-C levels in the body, thereby managing hyperlipidemia. The absence of PCSK9 in the body causes a person to have LDL-C levels of about 15 mg/dL. Therefore, PCSK9 inhibitors work towards restoring them to normal levels [4].

Gemcabene

Gemcabene plays a significant role in the management of hyperlipidemia. It downgrades the hepatic mRNA markers of inflammation [TNF-α, monocyte chemotactic protein-1 (MCP-1), macrophage inflammatory protein-1 beta (MIP-1β), chemokine receptor type 5 (CCR5), CCR2, nuclear factor-κB (NF-κB)], fibrosis [tissue inhibitors of metalloproteinases-1 (TIMP-1) and matrix metalloproteinase-2 (MMP-2)], lipid inflection, and lipogenesis [10]. These actions are essential because they prevent inflammation, steatosis, hepatocyte ballooning, and fibrosis development. Similarly, fibrates, niacin, and resins that bind bile acid are active in suppressing LDL-C.

## Conclusions

Hyperlipidemia is a life-threatening health condition that endangers the life of most patients. This condition is critical because it emanates from genomic make-up and hence occurs involuntarily. Environmental factors are also significant contributors to this condition. Other causes of hyperlipidemia emanate from secondary sources such as diet, drugs, and existing health conditions. All these causative agents represent a risk to human health; hence, reducing LDL-C remains a central therapeutic objective in the management of all hyperlipidemic patients. There are numerous management approaches to suppress LDL-C, including PCSK9 inhibitors, statins, gemcabene, bempedoic acid, and ezetimibe. All these therapeutic approaches aim at reducing the levels of LDL-C, and thereby controlling and managing hyperlipidemia. Despite the numerous approaches available for treating hyperlipidemia, people should observe their consumption rates, especially foods rich in oil. In short, while hyperlipidemia is manageable, human beings have a role in preventing its development.
